# Publisher intervention in editorial decision-making: when established editorial procedures no longer determine publication decisions

**DOI:** 10.1007/s00204-026-04560-3

**Published:** 2026-10-05

**Authors:** Jan G. Hengstler

**Affiliations:** Department of Toxicology, Leibniz Research Centre for Working Environment and Human Factors (IfADo), Ardeystr. 67, 44139 Dortmund, Germany

## Abstract

The credibility of scientific journals is often attributed to rigorous peer review and editorial independence. In reality, however, scientific publishing, like every human institution, is shaped by competing interests, institutional incentives, and errors of judgment. Its credibility therefore depends less on the presumed virtues of authors, reviewers, editors, and publishers than on transparent procedures, clearly defined responsibilities, and institutional arrangements that make departures from established standards visible and open to scientific scrutiny. This Editorial examines a revealing recent case in which publication of a Guest Editorial that had successfully completed the established editorial procedures of *Archives of Toxicology* was ultimately prevented by the Publisher. Rather than discussing the scientific merits of the underlying manuscript, the present analysis focuses on how the publication decision changed after the manuscript had already been formally accepted. The documented events show how the publication process was progressively compromised through a succession of additional procedural requirements, each introduced only after the preceding requirement had been fulfilled. As a consequence, publication no longer depended on completion of the journal’s published editorial process but on successive interventions outside that process. The case illustrates that transparent editorial procedures alone are insufficient to safeguard editorial integrity. Effective institutional self-correction requires a second, equally important safeguard: the guaranteed possibility that alleged departures from editorial procedures can themselves be subjected to unobstructed public scientific scrutiny through mechanisms such as Commentaries or Opinion Articles. In the present case, both safeguards were compromised simultaneously. The established editorial procedures were progressively displaced, while the very mechanism intended to permit public examination of alleged procedural failures was itself prevented from operating. The present analysis argues that departures from editorial procedures are not corrected automatically. They become self-correcting only when independent publication pathways remain available in which transparent editorial governance is preserved. The long-term credibility of scientific publishing therefore rests not primarily on confidence in individuals but on institutional structures that make procedural departures visible, increase the expected cost of abusing institutional power, and guarantee that alleged failures of governance can themselves be subjected to public scientific scrutiny. Transparency without the guaranteed possibility of such scrutiny remains incomplete.

## The foundations of scientific credibility

One of the most important questions in scientific publishing is what underlies the credibility of scientific journals—and, conversely, what may undermine it. The answer is unlikely to be found in the comforting assumption that authors, reviewers, editors or publishers are consistently free of personal interests, institutional loyalties, financial incentives or errors of judgment. They are not. Science is a human enterprise, and scientific publishing is no exception. Authors hope that their work will be accepted and well-received by the community, reviewers have their own scientific preferences and professional networks, editors are not immune to personal convictions, and publishers are commercial organizations operating under economic constraints and incentives rather than altruism. None of these sources of partiality are remarkable; indeed, it would be remarkable if it were otherwise. More than two thousand years ago, Cicero observed that *“nothing is so securely protected that it cannot be corrupted” (Cicero, 70 *BCE*).* Subsequent history over two millennia has provided little evidence that human nature has fundamentally changed. Institutions that depend primarily on the presumed virtue of those who administer them rarely remain robust over time.

Scientific publishing has not escaped the realities that shape other human institutions. Conflicts of interest, institutional loyalties, commercial incentives, personal ambitions, and professional vanity all remain part of everyday academic life. The question is therefore not how to eliminate these influences, which experience shows is unrealistic. The more practical question is how institutions can be organised so that such influences become visible, reviewable, contestable and, where necessary, correctable.

Transparency does not eliminate self-interest or poor judgement. Instead, its principal value comes from making editorial decisions visible and open to scrutiny. Transparency changes incentives. Behaviour that departs from established procedures is more likely to be recognised, questioned, publicly evaluated, and if necessary rectified. In this way, transparency increases the expected consequences of self-interested behaviour and thereby reduces its expected benefits. Institutions of scientific publishing should not be judged by whether they prevent every departure from established procedures; no institutional design can achieve that. They should instead be judged by how readily such departures become noticeable, how easily they can be questioned, and how effectively and effortlessly they can be corrected. If scientific publishing has one advantage over the Roman Senate, it is unlikely to be the superior virtue of the people involved. Its advantage may lie, or at least should lie, in the transparency of its procedures.

When journals publicly describe their editorial procedures, editorial decisions become visible and ideally understandable. Transparent editorial procedures are an essential prerequisite for accountable scientific publishing. Key elements should include defining the respective responsibilities of reviewers, editors and the publisher; explaining how manuscripts are evaluated; and communicating openly when established procedures have been modified. However, well-defined editorial procedures are not sufficient by themselves. Once departures become visible, scientific publishing also requires the availability of institutional mechanisms through which such departures can themselves be examined, criticised and, where appropriate, corrected.

One such mechanism is the possibility of publishing Commentaries and Opinion articles. Their function is not merely to express scientific opinions. They also provide an institutional forum in which real or perceived departures from established editorial procedures can themselves become the subject of public scientific review and discussion (Sachinidis et al. 2026). Indeed, the guaranteed possibility for scientists to publish Commentaries and Opinions whenever they believe that established editorial procedures have not been properly followed is among the most important of the mechanisms available for maintaining the integrity of the scientific record. Such public scrutiny does not eliminate bias, but it creates accountability. In institutional terms, transparency changes incentives. Decisions that depart from established practice or that are obviously unfair can no longer remain invisible. For more than two decades, *Archives of Toxicology* has regarded these principles of integrity and transparency as fundamental. Commentaries and Opinion Articles represent the views of their authors, not those of the Editors or the Publisher. Often, they criticize problematic procedures or decisions in our journal or at other places of the scientific world. During the past two decades, not a single Commentary or Opinion Article has been rejected by *Archives of Toxicology*, even if the Editors disagreed with the opinion expressed. This editorial policy was adopted not because we assumed exceptional virtue on the part of Editors but because we assumed precisely the opposite. Human interests do not disappear when individuals become editors. Our microcosm of scientific publishing is no more immune to personal interests, institutional loyalties or human ambition than the Roman Republic observed by Cicero. And—of course—Editors are not appointed because they are expected to agree with every published viewpoint. They are appointed because they are expected to recognize scientific quality. The guaranteed possibility to publish well-argued opinions with which Editors themselves disagree is not a weakness of editorial judgement but rather one of its clearest expressions.

It is precisely this procedural transparency that gives rise to credibility. Authors are more likely to submit their best work to journals whose editorial procedures are regarded as predictable, transparent, and consistently applied. Credibility, in other words, is not founded on trust in individuals; it is founded on confidence that the procedures are reliably applied and therefore deserve to be trusted.

For an Editor, credibility is among the journal’s most valuable assets—again, not as a consequence of personal virtue but because it determines the journal’s reputation, attractiveness, and long-term success. It is painstakingly acquired over many years. Every mistake or deviation from established editorial procedures should therefore be exceptional, but if they occur, they must be transparently discussed and, if necessary, openly criticized in the scientific community and rapidly corrected. Otherwise, the very mechanisms that create confidence in scientific publishing will rapidly erode. The value of institutional safeguards is rarely appreciated while they function smoothly. Their importance becomes fully apparent only when they are tested. The events described below (Table 1) are therefore not presented because of the scientific topic they concern. They are discussed because they provide an opportunity to examine how these institutional principles operate when they are placed under unusual strain.

**Table 1:** Overview of events

Date	Events
5 December 2025	*Regulatory Toxicology and Pharmacology* retracted the 25-year-old review by Williams et al. (2000) on the safety assessment of glyphosate
27 February 2026	A Commentary criticizing the retraction was submitted to *Regulatory Toxicology and Pharmacology*. The Co-Editor-in-Chief indicated that publication would only be considered if the manuscript were substantially shortened, to an extent that the authors considered incompatible with a meaningful discussion of the retraction. The manuscript was therefore withdrawn
3 March 2026	The manuscript was submitted to Archives of Toxicology as a Guest Editorial
5 March 2026	Manuscript accepted for publication by the Editors following completion of the journal’s established editorial process. Page proofs were prepared, returned by the authors and publication was scheduled
7 April 2026	**First procedural intervention:** Springer Nature Research Integrity requested external peer review although this is not part of the journal’s published procedures for Guest Editorials
27 April 2026	Two independent external reviewers recommended publication after minor revision and described the manuscript as a valuable scientific contribution
27 April 2026	Revised manuscript accepted by the Editors following revision in response to the reviewers’ comments and again forwarded for publication
28 April 2026	**Second procedural intervention:** Springer Nature Research Integrity requested revised competing-interest declarations for all authors
7 May 2026	Springer Nature confirmed that the revised competing-interest declarations satisfied the Research Integrity Group. The Editors again forwarded the manuscript for publication
27 May 2026	**Third procedural intervention:** Even though the Borgert et al. (2026), guest editorial had successfully passed the external peer-review and detailed additional information as to possible conflicts of interest including the more than 60 co-signatories was given, the Publishing Director of Springer Nature informed the Editors that they would not publish the manuscript, referring to external legal advice
9 June 2026	Manuscript submitted to EXCLI Journal
19 June 2026	Accepted for publication following the journal’s normal editorial process
14 July 2026	Published in *EXCLI Journal*

**Overall pattern:** Each additional requirement was fulfilled before a new procedural requirement was introduced. Publication was ultimately prevented only after all requested steps had been completed

## The case study—a recent stress test of editorial governance

The institutional principles discussed above are not merely theoretical. They are occasionally tested under circumstances that reveal how editorial governance functions in practice. One such case has recently arisen under unusual circumstances and attracted considerable attention within the scientific community and beyond. For the first time in the history of *Archives of Toxicology*, publication of a Guest Editorial that had successfully completed the journal’s review process was prevented by the Publisher (Table 1). The submitted Guest Editorial concerned the recent retraction of a highly cited review article published in 2000 in *Regulatory Toxicology and Pharmacology* (Williams et al. 2000), which had presented a toxicological risk assessment of the herbicide glyphosate. The retraction itself (Williams et al. 2025) generated substantial scientific discussion because it concerned a publication that was widely cited in the toxicological literature for more than two decades. The authors of the Guest Editorial argued that the retraction was scientifically and procedurally unjustified and advanced a series of specific scientific and procedural arguments in support of that position. Among other points, they considered it inappropriate that the absence of a response from Professor Gary M. Williams was cited in the Retraction Notice, arguing that Professor Williams—one of the pioneers of modern genetic toxicology—was by then approaching ninety years of age and no longer realistically able to participate in such correspondence. They further argued that criticism in the Retraction Notice concerning the omission of unpublished studies was not justified since such studies were not available for inspection by the authors at the time the original review was prepared. Finally, they challenged the allegation of ghostwriting, noting that this assertion had previously been examined without finding evidence to substantiate the claim.

Whether these arguments are ultimately convincing is not the subject of the present Editorial. Scientists may reasonably agree or disagree with the conclusions reached by the authors of the Guest Editorial; indeed, that is precisely why scientific journals publish Commentaries, Letters, and Guest Editorials. Their purpose is not to establish scientific consensus but to provide a forum in which scientific arguments can be presented and vigorously discussed. The editorial evaluation of such contributions serves a different purpose. For Commentaries and Guest Editorials, the mandate is not to arbitrate scientific truth or to require editorial agreement with the authors’ conclusions. Rather, it is to ensure that arguments are presented logically, responsibly, and in a form that allows the scientific community to evaluate them critically.

## The established editorial process

The manuscript of the Guest Editorial entered the *Archives of Toxicology* submission portal through the established process for Guest Editorials and Commentaries. According to the journal’s published editorial procedures, manuscripts in this category are evaluated by the responsible Editors and, unlike original research articles, are not subjected to external peer review. The Editors evaluated the manuscript in accordance with these published principles not to determine whether the Editors agreed with the scientific opinions expressed but rather to verify that the manuscript fulfilled the editorial requirements for this article type. The Editors concluded that the manuscript presented a coherent scientific argument, that factual assertions and opinions were clearly distinguishable, that no personal attacks or unsupported allegations had been made, and that the manuscript represented a reasonable contribution to scientific debate on the topic—which was the appropriateness of the retraction process and not the scientific content of the retracted paper. Because the manuscript addressed a highly controversial topic with potential public implications, the Editors additionally sought independent legal advice from an experienced media lawyer. This assessment likewise identified no legal obstacle to publication. The manuscript was therefore accepted for publication by the Editorial Board. At this point, the manuscript had successfully completed the editorial evaluation required under the journal’s published procedures for reviewing Guest Editorial manuscripts.

## The first departure from established procedures

The editorial process did not conclude with the Editorial Board’s decision to accept the manuscript. By this stage, the manuscript had already been formally accepted for publication. Page proofs had been prepared, sent to the authors, corrected, and returned. Under the journal’s established editorial procedures, the next step would ordinarily have been publication.

Instead, the Editors received an email from a Senior Publishing Manager at Springer Nature informing them that the manuscript had been referred to Springer Nature’s Research Integrity Group. According to the communication, the Group requested that the manuscript undergo external peer review, although the journal’s published editorial procedures do not require external review for Guest Editorials or Commentaries. The Editors asked why a departure from the journal’s published procedures had become necessary after the manuscript had already completed the editorial process. They were informed that the manuscript concerned a politically sensitive topic. The Editors were unable to understand why the political sensitivity of a topic, in itself, necessitated any modification of the journal’s published editorial procedures or why additional scientific peer review would be expected to address what had been described as a political concern.

Nevertheless, the Editors agreed to this departure from the journal’s established procedures. They did not consider additional peer review necessary under the journal’s editorial policy. However, they also saw no disadvantage in obtaining an independent external scientific assessment if this would remove any possible suggestion that the manuscript had not been evaluated with sufficient rigor because of its controversial subject matter. The Editors therefore regarded this additional round of review not as a replacement of the established editorial process but as an additional safeguard.

Two independent subject matter experts were invited to review the manuscript. Both reviewers considered the manuscript as scientifically sound and worthwhile contribution to an important scientific debate and recommended publication after minor revision.

The authors revised the manuscript accordingly. During this process, the Publisher additionally requested disclosure of the identities of the reviewers. This represented a further departure from the journal’s established editorial practice, under which reviewer identities had always remained confidential. Only after obtaining the reviewers’ explicit consent did the Editors comply with this request.

At this point, every additional requirement introduced after completion of the journal’s normal editorial process had been addressed. The additional peer review had been completed. The scientific reviewers’ comments had been implemented. Both reviewers recommended publication.

Under the journal’s published editorial procedures, publication would ordinarily have followed.

It did not.

## When fulfilling one extraordinary requirement led to another

Completion of the additional peer review did not bring the editorial process to an end. Instead, the Editors received a further communication from Springer Nature informing them that the Springer Nature Research Integrity Group had requested revision of the manuscript’s declarations of competing interests. The manuscript already contained detailed competing-interest declarations in accordance with the journal’s established requirements. The additional request did not question the existence of these declarations but sought a different format together with further author-specific information. Because the manuscript had more than sixty co-authors, implementing these changes required extensive additional correspondence with all authors and considerable editorial effort. The Editors regarded careful disclosure of competing interests as an entirely legitimate aspect of scientific publishing. Although these additional requirements went beyond the journal’s normal editorial procedures, they again sought to accommodate the request. Their objective remained the same: to ensure that every concern raised during the editorial process had been addressed and that publication could then proceed without further delay.

The revised competing-interest declarations were obtained, and the Editors concluded that all requested revisions had been completed satisfactorily. The Editors subsequently received confirmation from Springer Nature that the Research Integrity Group was satisfied with the revised competing-interest declarations.

At this stage, every additional requirement that had been introduced after completion of the journal’s normal editorial process had been addressed to the satisfaction of both the Editors and the Springer Nature Research Integrity Group.

Under the journal’s published editorial procedures, publication would ordinarily have followed.

Once again, it did not.

Instead, a further intervention occurred.

## When legal advice became the justification for a publisher’s decision

The next development differed fundamentally from those preceding it. Until this stage, every intervention had introduced an additional procedural requirement that could, at least in principle, be addressed by the Editors and the authors. The next intervention was of a different nature. It no longer concerned the scientific evaluation of the manuscript or the completion of further editorial requirements. Instead, it concerned the authority to determine whether publication would take place at all.

The Editors subsequently received an email from the Publishing Director of Springer Nature informing them that an external legal assessment had been obtained from a specialist media lawyer. The accompanying legal review was made available to the Editors on a confidential basis together with the instruction that it should not be shared with the authors or with third parties.

The legal review itself adopted a differentiated approach. Rather than concluding that the manuscript contained unlawful statements or that publication would necessarily expose the Publisher to legal liability, it examined certain text passages from the perspective of legal risk management. Among other observations, it considered whether certain individuals needed to be identified by name, whether some formulations might be expressed differently, and whether alternative wording could further reduce potential legal exposure while preserving the substance of the article. Such observations are entirely consistent with the role of specialist legal counsel. Their task is to identify potential legal vulnerabilities and, where appropriate, suggest formulations that might further reduce legal risk.

The subsequent communication from Springer Nature, however, did not present the legal assessment in these differentiated terms. Instead, the Editors were informed that the legal advice indicated *“a high risk of defamation”* that would remain even if the manuscript were revised or shortened. The communication concluded with the following decision:

“Considering the legal advice received and our responsibilities as a publisher, Springer Nature will not publish this article.”

The present Editorial does not question the legitimacy of obtaining legal advice. Publishers have every reason to seek specialist legal expertise whenever they consider that legal risks may exist. The institutional question begins only after such advice has been obtained. How should legal assessments be incorporated into editorial decision-making? Who ultimately decides whether the identified legal concerns require revision, further discussion with the authors, or complete withdrawal of a manuscript from publication?

In the present case, that decision was not made within the journal’s editorial process.

It was not the decision of the handling Editors.

It was not the decision of the Editorial Board.

It was not the recommendation of the external reviewers.

It was not the recommendation of a specialist media lawyer.

It was solely the decision of the Publisher.

A further feature of the process deserves attention. Although the publication decision was explicitly based on the legal assessment, the Editors were instructed that the legal review itself was confidential and should not be shared with the authors or with third parties. The authors therefore received the publication decision, but not the document on which that decision was said to rest. Whether such an approach is compatible with the broader principles of transparency discussed above is a question to which the present Editorial will return.

## When the working hypothesis collapsed

Throughout the preceding stages, the Editors had attempted to reconcile the Publisher’s requests with their responsibilities towards the authors. They proceeded on the assumption that the established editorial process remained fundamentally intact and that each additional request represented an effort to strengthen its robustness rather than to replace it. This interpretation was consistent with normal editorial practice and explained why the Editors repeatedly sought to accommodate successive requests.

The final communication from Springer Nature no longer supported that interpretation. Publication was prohibited despite completion of every procedural step that had been requested after the manuscript had already been accepted. As in scientific investigation, when observations are no longer compatible with the working hypothesis, it is the hypothesis—not the observations—that must be reconsidered. The Editors therefore recognised that the successive interventions could no longer be understood simply as additional components of the established editorial process.

From that point onwards, continuing to present the Publisher’s decision as though it formed part of the journal’s normal editorial process would itself have conflicted with the principles of procedural transparency discussed in the first part of this Editorial. The Editors therefore concluded that the authors should be informed openly about the sequence of events and about the respective responsibilities of the Editors and the Publisher, even though this required abandoning the previous strategy of attempting to resolve the matter internally. Only on the basis of such transparency could the authors decide how they wished to proceed.

The Editors further concluded that the immediate handling of a single manuscript had become secondary to a more fundamental responsibility: protecting the procedural integrity on which the journal’s long-term credibility depends. Scientific journals do not lose credibility because difficult editorial decisions sometimes become necessary. They lose credibility when the procedures by which those decisions are reached become opaque, when established responsibilities are displaced without transparency, or when it is no longer possible for authors, reviewers and readers to understand how publication decisions have actually been reached.

## An alternative editorial pathway

The events described above required the Editors to reconsider not only the handling of a single manuscript but also their broader responsibilities towards authors and the scientific community. Once it had become clear that the publication decision no longer resulted from the editorial procedures publicly described by the journal, preserving procedural transparency became the overriding consideration.

Fortunately, preparations had already been made for situations in which editorial independence might no longer be fully preserved. Some of the Editors had previously established an independent scientific journal operating outside the structures of a commercial publishing house. The journal follows a conventional editorial model based on independent editorial assessment and external peer review where appropriate. It is managed by academic Editors, does not charge article processing fees, and provides unrestricted access to all published articles.

After being informed about the sequence of events and the respective responsibilities of the Editors and the Publisher, the authors decided to submit their manuscript to this journal. The manuscript entered the journal through its normal editorial process, underwent editorial evaluation and external peer review, was revised in response to the reviewers’ comments, and was subsequently accepted for publication. It has since been published (Borgert et al. 2026).

At the time of writing, none of the legal consequences anticipated by Springer Nature have materialised. This observation is not presented as evidence that legal concerns should be disregarded. Legal risk is often difficult to assess, and different publishers may reasonably arrive at different conclusions. The point is more limited. The existence of an alternative editorial structure ensured that the scientific arguments contained in the manuscript remained available for critical evaluation by the scientific community rather than being excluded from scientific discussion altogether.

From an institutional perspective, this illustrates an important principle. Scientific publishing benefits when editorial independence is not concentrated within a single organisational structure. The availability of independent editorial pathways provides an important mechanism of self-correction whenever authors, editors or publishers reach fundamentally different conclusions about the appropriate handling of a manuscript.

## Institutional self-correction in scientific publishing

The present case raises questions that extend far beyond a single publisher, a single journal, or a single manuscript. It invites reflection on how scientific publishing should be organised if editorial independence, procedural transparency, and institutional accountability are to remain credible in the future.

The first conclusion is straightforward. Clearly defined editorial procedures are essential, but they are not sufficient. Their value depends entirely on whether they are applied consistently and without external interference in editorial decision-making. Procedures that may be altered during the editorial process without transparent justification no longer provide the predictability on which authors, reviewers and readers rely. Editorial independence is therefore not protected simply by publishing editorial policies. It depends on whether those policies continue to reliably govern editorial decisions when they are most severely tested.

Publishers possess the institutional power to intervene in editorial procedures. In practice, they may therefore alter or replace a journal’s established decision-making process. The central question is therefore not whether such power and authority exist, but which institutional mechanisms are available to increase the cost of their misuse. Such self-correcting mechanisms are essential to ensure that departures from established procedures become visible, and allow such departures to be openly scrutinised by the scientific community.

Scientific publishing does not correct itself automatically. History provides many examples in which institutional monopolies have suppressed rather than facilitated scientific debate. Self-correction becomes possible only where alternative (editorial) structures exist and are able to compete transparently for the confidence of authors and readers, and for the manuscripts with the best results. Its long-term integrity does not depend on the flawless functioning of every individual journal. Rather, it depends on the continued existence of alternative editorial structures that remain genuinely independent. If one editorial pathway no longer provides the procedural safeguards expected by authors and readers, scientific self-correction remains possible only if alternative journals continue to exist in which those safeguards are preserved. In this sense, editorial independence is not merely a property of individual journals; it is a property of the scientific publishing ecosystem as a whole. This, in turn, requires genuine diversity of independent competing publication systems offering transparent procedures, editorial independence, and scientific credibility. Such competition creates an evolutionary advantage for journals that consistently uphold these principles. By contrast, the concentration of scientific publishing in the hands of a single dominant publisher—or a small number of publishers whose policies and practices become increasingly aligned—would inevitably weaken these corrective mechanisms. Institutional diversity is therefore not merely a matter of fashion or market structure. It is one of the safeguards on which the long-term integrity of scientific publishing depends.

This mechanism operates without central coordination. Authors submit their strongest work to journals whose reputation is outstanding and whose editorial procedures they consider to be transparent, predictable, and intellectually independent. Readers place greatest confidence in journals whose publication decisions they regard as justified, understandable, and procedurally consistent. Reputation is therefore not created by declarations of principle but by their sustained and constant application in practice. Over time, this reliability creates powerful incentives. Journals that consistently protect procedural integrity strengthen their scientific standing. Journals that repeatedly depart from their own published procedures inevitably place that standing at risk.

## The future of the journal

The observations presented in this Editorial are not directed solely at one publisher or one journal. They apply equally to every scientific journal, including our own. The present case threatens two institutional principles that are essential for the credibility of scientific publishing. First, the journal’s published editorial procedures were set aside in contravention to editorial autonomy and without transparent procedural justification. The events described in this Editorial represent the first occasion in the history of *Archives of Toxicology* on which publication of a manuscript that had successfully completed the journal’s established editorial process was ultimately prevented by the Publisher through a sequence of serial, extraordinary interventions that departed from the journal’s published editorial procedures and progressively displaced the procedural transparency on which the journal’s editorial governance and reputation had been built. The second issue is potentially even more important. As discussed in the first part of this Editorial, the guaranteed possibility of publishing Commentaries and Opinion articles constitutes one of the principal mechanisms by which scientific publishing is able to correct procedural failures. In the present case, this mechanism itself became the object of intervention. The Guest Editorial had been submitted precisely because its authors believed that established editorial procedures had not been properly followed in connection with the retraction of a previously published article. By preventing publication of that Guest Editorial after completion of the journal’s editorial process, the intervention not only departed from the journal’s established procedures but simultaneously disabled one of the core institutional safeguards through which such departures can normally be examined and openly discussed by the scientific community.

The motivations underlying such interventions deserve careful examination since departures from established procedures rarely occur without institutional incentives or conflicting interests. Identifying those incentives, however, lies beyond the scope of the present analysis.

Whether the present case will remain an isolated exception or prove to reflect a more fundamental change that recurs in the future, on a regular or even occasional basis, cannot presently be known. What can already be stated, however, is that the future credibility of *Archives of Toxicology* will depend on whether procedural transparency can be fully restored, whether responsibility for editorial decisions once again becomes clearly identifiable, and whether the events described here are subjected to the same openness and critical scrutiny that scientific journals routinely expect of the research they publish.

For this reason, the present Editorial should not be regarded as the conclusion of the discussion but as its beginning. Scientific journals exist for the scientific community. Their long-term legitimacy cannot be determined solely by editors or publishers. It depends ultimately on the judgement of authors, reviewers and readers. Their willingness to scrutinise editorial procedures, to question departures from established practice where appropriate, and to participate openly in this discussion without any interference from non-editorial entities constitutes one of the most effective safeguards of editorial independence available to scientific publishing.

The patient described in this case is therefore not beyond recovery. However, recovery cannot be achieved by concealing the diagnosis. As in every field that aspires to rigorous scientific standards, the first prerequisite for effective treatment is an accurate description of the underlying pathology.

## Data Availability

No datasets were generated or analysed during the current study.

## References

[CR1] Borgert CJ, Mohammad A, Ansell JM (2026) Retraction of the landmark glyphosate safety publication by Williams, Kroes and Munro (2000) should be reversed. EXCLI J 25:1107–1116 10.17179/excli2026-9644 PMC13402740 42517087

[CR2] Cicero, In Verrem, Actio Prima, 70 BC, Nihil esse tam sanctum quod non violari, nihil tam munitum quod non expugnari pecunia possit

[CR3] Sachinidis A, Albrecht W, Hengstler JG (2026) Structured, evidence-based debate in science: introducing a new forum. EXCLI J 19(25):846–847. 10.17179/excli2026-9618 PMC13365114 42454333

[CR4] Williams GM, Kroes R, Munro IC (2000) Retracted: safety evaluation and risk assessment of the herbicide Roundup and its active ingredient, glyphosate, for humans. Regul Toxicol Pharmacol 31(2):117–165. 10.1006/rtph.1999.1371 10854122

[CR5] Williams GM, Kroes R, Munro IC (2025) Retraction notice to “Safety evaluation and risk assessment of the herbicide roundup and its active ingredient, glyphosate, for humans” [Regul. Toxicol. Pharm. 31 (2000) 117–165]. Regul Toxicol Pharmacol 4: 106006. 10.1016/j.yrtph.2025.106006 41428335

